# Association Between Meteorological Conditions and Acute Myocardial Infarction (AMI) Incidence: A Seasonal Analysis Using the Oita AMI Registry

**DOI:** 10.7759/cureus.86686

**Published:** 2025-06-24

**Authors:** Shinpei Ono, Hidefumi Akioka, Hiroki Sato, Takuto Zaizen, Shuichiro Yamauchi, Hiroyuki Kodama, Hidekazu Kondo, Tetsuji Shinohara, Kunio Yufu, Naohiko Takahashi

**Affiliations:** 1 Department of Cardiology and Clinical Examination, Faculty of Medicine, Oita University, Yufu, JPN; 2 Disaster Management Center, Oita University Hospital, Yufu, JPN

**Keywords:** acute myocardial infarction, atmospheric pressure, frequent onset days, oita ami registry, weather conditions

## Abstract

Background

Weather is recognized as an environmental risk factor for acute myocardial infarction (AMI). This study explores the meteorological factors linked to a higher incidence of AMI.

Methods and results

The study involved 403 patients from the Oita AMI Registry who were admitted to 20 medical facilities with an AMI diagnosis between April 2012 and September 2013. We defined “frequent onset days” (F-days) as days when three or more patients were admitted for AMI, in contrast to "non-frequent onset days” (non-F-days), which were days with fewer than three admissions. This period was categorized into four seasons: spring, summer, autumn, and winter. The meteorological data used in this study came from the Japan Meteorological Agency. During autumn, F-days were associated with a significantly older patient population (77.4 ± 11.7 years vs. 68.3 ± 13.8 years, p = 0.02) and a higher population of female patients (57.1% vs. 17.4%, p = 0.003) compared to non-F-days. Additionally, the body mass index was significantly lower on F-days compared to non-F days (21.5 ± 3.5 kg/m² vs. 23.6 ± 3.2 kg/m², p = 0.03). In summer, the average atmospheric pressure was significantly higher on F-days than on non-F-days for day-1 (1009.8 ± 2.8 hPa vs. 1007.1 ± 3.7 hPa, p = 0.03) and day-2 (1010.1 ± 2.7 hPa, compared to 1007.2 ± 3.7 hPa, p = 0.02). In autumn, among the variables that showed significant differences, only female patients remained independently associated with the risk of F-days in the multivariate analysis (p = 0.007).

Conclusions

Our research indicated that elevated atmospheric pressure during summer was associated with a higher occurrence of AMI on days with frequent onset of cardiac events. In autumn, women were independently associated with a higher occurrence of AMI on such days.

## Introduction

Earlier research indicates that acute myocardial infarction (AMI) occurs with seasonal variation [1,2]. AMI frequency has been linked to cold winter temperatures [3,4], high summer temperatures and humidity, and low atmospheric pressure [5]; however, the findings have been inconsistent. This study examined the incidence of AMI in Oita Prefecture, exploring the link between meteorological factors and AMI frequency throughout the four seasons. By pinpointing the days with high onset frequencies, we aim to offer fresh insights into how local meteorological conditions influence the onset of AMI. This study aimed to explore seasonal meteorological factors associated with the incidence of AMI in Oita Prefecture, focusing on observational patterns without implying causality.

## Materials and methods

Study design and population

We conducted a retrospective observational study using data from the Oita AMI Registry. This registry prospectively and consecutively enrolled 403 patients admitted with AMI to 20 hospitals located in Oita Prefecture, Japan, from April 2012 to September 2013. Details of the Oita AMI Registry have been described elsewhere [6,7].

Seasonal and meteorological classification

The registry period was categorized into four seasons: spring (March, April, May), summer (June, July, August), autumn (September, October, November), and winter (December, January, February). We defined frequent onset days (F-days) as those with three or more AMI admissions, while non-frequent onset days (non-F-days) were classified as having fewer than three admissions [8].

Meteorological data were obtained from the Japan Meteorological Agency, specifically from the Oita City weather station, which is representative of the major urban center in the region. Cardiac catheterization facilities in Oita Prefecture are concentrated mainly in Oita City and Beppu City. Based on the locations of the facilities where treatments were performed, meteorological conditions were primarily derived from data collected in Oita City. We examined daily maximum and minimum temperatures, intraday temperature variation (defined as the difference between maximum and minimum temperatures), and average atmospheric pressure and humidity. These meteorological variables were assessed on the day of onset (day-0), as well as one day (day-1), and two days (day-2) prior. Meteorological data were matched to each patient’s AMI onset date based on the registry records. The date and time of AMI onset were determined by the attending physician. There were no missing data in meteorological or onset variables; all cases were included without exclusion or imputation. In this study, “high atmospheric pressure” was defined as a significantly higher pressure on F-days compared to non-F-days during the same season.

Diagnosis definitions

This study incorporated all patients diagnosed with AMI, which includes ST-elevation myocardial infarction (STEMI) and non-ST-elevation myocardial infarction (non-STEMI), into the registry. The diagnostic criteria for AMI were based on the joint guidelines set forth by the European Society of Cardiology and the American College of Cardiology in 2000 [9] and the World Health Organization criteria [10].

Ethical standards

The study procedures involving human participants adhered to the ethical standards set by each institutional research committee, the 1964 Declaration of Helsinki, and its subsequent amendments or similar ethical guidelines standards. This study was approved by the Medical Ethics Committee of Oita University Hospital (Approval No. 529). Written informed consent was obtained from all participants prior to their inclusion in the registry.

Statistical analysis

Continuous variables are expressed as means ± standard deviation (SD). Categorical variables are presented as counts and percentages. Group differences for continuous variables were evaluated using the student’s t-test, and for categorical variables, the chi-square test was applied. Variables with a P value of <0.05 in the univariate analysis, including sex, age, and body mass index (BMI), were included in the multivariate logistic regression model to identify factors independently associated with F-days in autumn.

All statistical analyses were performed using JMP version 14 (SAS Institute, Cary, NC, USA) on Windows 11 (Microsoft, Redmond, WA, USA). A p-value of <0.05 was regarded as statistically significant.

## Results

Baseline characteristics

Table 1 presents the baseline characteristics of the entire study population. Tables 2-5 show the baseline characteristics stratified by season: spring, summer, autumn, and winter, respectively. In autumn, the occurrence of AMI on F-days corresponded with a higher ratio of older (77.4 ± 11.7 years vs. 68.3 ± 13.8 years, p = 0.02) and female patients (57.1% vs. 17.4%, p = 0.003) as compared to non-F-days (Table 4). Additionally, the BMI was significantly lower on F-days compared to non-F days (21.5 ± 3.5 kg/m² vs. 23.6 ± 3.2 kg/m², p = 0.03). Baseline characteristics were described only for autumn, as no significant differences were observed in other seasons.

**Table 1:** The seasonal meteorological conditions in Oita Prefecture All values are presented as mean ± standard deviation.

	Spring	Summer	Autumn	Winter
Average maximum temperature (℃)	20.6±4.6	30.4±4.5	24.4±5.3	10.8±2.7
Average minimum temperature (℃)	11.4±4.8	23.0±2.8	16.2±5.8	2.6±2.9
Average intraday temperature difference (℃)	9.2±3.7	7.4±2.7	8.2±2.5	8.2±2.4
Average atmospheric pressure (hPa)	1012.2±5.4	1007.2±3.6	1013.5±4.4	1020.2±4.7
Average humidity (%)	64.7±12.6	75.7±8.7	68.0±10.7	65.3±11.9

**Table 2:** Basic characteristics of AMI patients in spring Continuous variables are presented as mean ± standard deviation and compared using the t-test. Categorical variables are presented as frequencies and percentages and compared using the chi-square test. Test statistics are reported as t-values for continuous variables and chi-square values for categorical variables. *p < 0.05 was considered statistically significant. BMI: Body Mass Index; AMI: Acute Myocardial Infarction

	F-days	Non-F-days	Statistical test value	p-value
Female (%)	5/31 (16.1%)	29/94 (30.1%)	2.75	0.10
Age (years)	74.3±9.1	72.1±12.8	-0.93	0.35
BMI (kg/m^2^)	22.9±2.9	24.2±4.1	1.52	0.13
Coronary risk factors				
Current smoker	13/31 (41.9%)	32/94 (34.0%)	0.56	0.43
Hypertension	24/31 (77.4%)	70/94 (74.5%)	0.59	0.44
Diabetes mellitus	10/31 (32.3%)	33/94 (35.1%)	0.77	0.08
Dyslipidemia	18/31 (58.1%)	53/94 (56.4%)	0.03	0.87

**Table 3:** Basic characteristics of AMI patients in summer Continuous variables are presented as mean ± standard deviation and compared using the t-test. Categorical variables are presented as frequencies and percentages and compared using the chi-square test. Test statistics are reported as t-values for continuous variables and chi-square values for categorical variables. *p < 0.05 was considered statistically significant. BMI: Body Mass Index; AMI: Acute Myocardial Infarction

	F-days	Non-F-days	Statistical test value	p-value
Female (%)	10/28 (35.7%)	27/102 (26.5%)	0.89	0.34
Age (years)	72.6±11.2	70.0±11.6	-1.05	0.29
BMI (kg/m^2^)	24.4±3.4	23.1±3.7	-1.51	0.13
Coronary risk factors				
Current smoker	6/28 (21.4%)	36/102 (35.3%)	2.04	0.15
Hypertension	22/28 (78.6%)	73/102 (71.6%)	0.56	0.45
Diabetes mellitus	14/28 (50.0%)	35/102 (34.3%)	2.25	0.13
Dyslipidemia	18/28 (64.3%)	62/102 (60.8%)	0.11	0.74

**Table 4:** Basic characteristics of AMI patients in autumn Continuous variables are presented as mean ± standard deviation and compared using the t-test. Categorical variables are presented as frequencies and percentages and compared using the chi-square test. Test statistics are reported as t-values for continuous variables and chi-square values for categorical variables. *p < 0.05 was considered statistically significant. BMI: Body Mass Index; AMI: Acute Myocardial Infarction

	F-days	Non-F-days	Statistical test value	p-value
Female (%)	8/14 (57.1%)	12/69 (17.4%)	8.78	0.003*
Age (years)	77.4±11.7	68.3±13.8	-2.32	0.02*
BMI (kg/m^2^)	21.5±3.5	23.6±3.2	2.15	0.03*
Coronary risk factors				
Current smoker	3/14 (21.4%)	25/69 (36.2%)	1.22	0.27
Hypertension	10/14 (71.4%)	39/69 (56.5%)	1.11	0.29
Diabetes mellitus	5/14 (35.7%)	21/69 (32.1%)	0.15	0.70
Dyslipidemia	7/14 (50.0%)	46/69 (67.9%)	1.36	0.24

**Table 5:** Basic characteristics of AMI patients in winter Continuous variables are expressed as mean ± standard deviation, and categorical variables are presented as frequencies and percentages. BMI: Body Mass Index; AMI: Acute Myocardial Infarction

	F-days	Non-F-days	p-value
Male/Female	-	13/65 (20%)	-
Age (years)	-	69.0±12.5	-
BMI (kg/m^2^)	-	24.0±3.8	-
Coronary risk factors			-
Current smoker	-	23/65 (35.4%)	-
Hypertension	-	49/65 (75.4%)	-
Diabetes mellitus	-	25/65 (38.5%)	-
Dyslipidemia	-	29/65 (44.6%)	-

Table 6 illustrates the comparison of spring weather on F-days and non-F-days. No significant differences were found in patient characteristics or meteorological variables between F-days and non-F-days throughout spring.

**Table 6:** Comparison of meteorological conditions between F-days and non-F-days in spring Continuous variables are expressed as mean ± standard deviation and compared using the independent two-sample t-test. Test statistics (t-values) and corresponding p-values are reported. *p < 0.05 was considered statistically significant.

Meteorological factors	F-days (n=10)	Non-F-days (n=143)	t-value	p-value
Maximum temperature (℃)				
Day-2	21.0±5.2	20.6±4.5	-0.26	0.80
Day-1	20.1±4.6	20.7±4.5	0.43	0.66
Day-0	20.3±5.6	20.8±4.4	0.31	0.76
Minimum temperature (℃)				
Day-2	12.2±5.0	11.2±4.7	-0.70	0.49
Day-1	10.4±4.9	11.4±4.8	0.66	0.51
Day-0	10.8±3.3	11.5±4.9	0.45	0.64
Intraday temperature difference (℃)				
Day-2	8.8±2.2	9.5±3.7	0.59	0.56
Day-1	9.7±3.9	9.3±3.6	-0.33	0.74
Day-0	9.5±3.3	9.3±3.7	-0.22	0.83
Atmospheric pressure (hpa)				
Day-2	1010.2±6.8	1012.3±5.3	1.20	0.23
Day-1	1011.9±3.4	1012.2±5.5	0.13	0.90
Day-0	1012.9±3.2	1012.1±5.5	-0.46	0.65
Humidity (%)				
Day-2	62.0±10.6	64.3±12.6	0.55	0.58
Day-1	63.6±11.3	64.4±12.7	0.19	0.85
Day-0	63.7±8.0	64.7±12.8	0.25	0.81

Table 7 compares summer weather conditions between F-days and non-F-days. On day-1, the average atmospheric pressure was significantly higher on F-days than on non-F-days (1009.8 ± 2.8 hPa vs. 1007.2 ± 3.7 hPa, p = 0.03). Similarly, on day-2, the pressure on F-days was 1010.1 ± 2.7 hPa as compared to 1007.2 ± 3.7 hPa on non-F-days (p = 0.02).

**Table 7:** Comparison of meteorological conditions between F-days and non-F-days in summer Continuous variables are expressed as mean ± standard deviation and compared using the independent two-sample t-test. Test statistics (t-values) and corresponding p-values are reported. *p < 0.05 was considered statistically significant.

Meteorological factors	F-days (n=9)	Non-F-days (n=175)	t-value	p-value
Maximum temperature (℃)				
Day-2	29.4±4.9	30.2±4.6	0.54	0.59
Day-1	28.7±5.5	30.4±4.5	1.07	0.30
Day-0	29.8±5.7	30.4±4.4	0.37	0.71
Minimum temperature (℃)				
Day-2	21.7±3.7	22.9±2.9	1.15	0.25
Day-1	22.0±3.4	23.0±2.9	0.99	0.32
Day-0	22.0±3.4	23.1±2.8	1.10	0.29
Intraday temperature difference (℃)				
Day-2	7.7±3.0	7.4±2.8	-0.32	0.75
Day-1	6.8±3.2	7.4±2.7	0.67	0.50
Day-0	7.8±3.1	7.3±2.7	-0.50	0.61
Atmospheric pressure (hpa)				
Day-2	1010.1±2.7	1007.2±3.7	-2.34	0.02*
Day-1	1009.8±2.8	1007.1±3.7	-2.14	0.03*
Day-0	1008.7±2.8	1007.1±3.6	-1.31	0.19
Humidity (%)				
Day-2	75.4±7.1	75.7±8.8	0.08	0.94
Day-1	78.1±8.6	75.6±8.6	-0.85	0.40
Day-0	76.3±7.8	75.7±8.7	-0.20	0.84

Table 8 compares autumn weather conditions between F-days and non-F-days. During the autumn season, no significant differences in meteorological variables were observed between the two groups.

**Table 8:** Comparison of meteorological conditions between F-days and non-F-days in autumn Continuous variables are expressed as mean ± standard deviation and compared using the independent two-sample t-test. Test statistics (t-values) and corresponding p-values are reported. *p < 0.05 was considered statistically significant.

Meteorological factors	F-days (n=4)	Non-F-days (n=117)	t-value	p-value
Maximum temperature (℃)				
Day-2	26.0±5.6	24.7±5.4	-0.47	0.64
Day-1	26.3±6.4	24.5±5.4	-0.64	0.52
Day-0	25.3±8.1	24.4±5.3	-0.34	0.73
Minimum temperature (℃)				
Day-2	16.5±8.7	16.5±5.8	0.008	0.99
Day-1	18.2±6.2	16.3±5.9	-0.62	0.53
Day-0	16.7±9.0	16.1±5.8	-0.20	0.83
Intraday temperature difference (℃)				
Day-2	9.5±3.2	8.2±2.6	-1.00	0.32
Day-1	8.1±0.2	8.2±2.6	0.08	0.94
Day-0	8.6±1.1	8.2±2.6	-0.26	0.80
Atmospheric pressure (hpa)				
Day-2	1013.7±2.7	1013.2±4.6	-2.11	0.83
Day-1	1013.8±3.5	1013.3±4.6	-0.21	0.84
Day-0	1014.6±2.2	1013.5±4.5	-0.47	0.64
Humidity (%)				
Day-2	67.2±7.7	67.8±10.6	0.10	0.92
Day-1	66.3±13.1	67.9±10.6	0.30	0.76
Day-0	68.8±9.0	67.8±10.6	-0.17	0.86

Table 9 presents the winter data analysis. During the winter season, there were non-F-days, indicating that no frequent onset days occurred in this season. Therefore, no F-day data for winter are available. These seasonal analyses showed that meteorological conditions on F-days and non-F-days differed significantly only in atmospheric pressure during summer.

**Table 9:** Comparison of meteorological conditions between F-days and non-F-days in winter All values are presented as mean ± standard deviation.

Meteorological factors	F-days (n=0)	Non-F-days (n=90)	p-value
Maximum temperature (℃)			
Day-2	-	10.8±2.7	-
Day-1	-	10.8±2.7	-
Day-0	-	10.8±2.7	-
Minimum temperature (℃)			
Day-2	-	2.6±2.9	-
Day-1	-	2.5±2.9	-
Day-0	-	2.6±2.9	-
Intraday temperature difference (℃)			
Day-2	-	8.2±2.4	-
Day-1	-	8.2±2.4	-
Day-0	-	8.2±2.4	-
Atmospheric pressure (hpa)			
Day-2	-	1020.3±4.7	-
Day-1	-	1020.2±4.7	-
Day-0	-	1020.2±4.7	-
Humidity (%)			
Day-2	-	65.1±11.8	-
Day-1	-	65.5±11.8	-
Day-0	-	65.3±11.8	-

Table 10 presents the results of the multivariate logistic regression analysis examining factors associated with F-days in autumn. Female patients were significantly associated with an increased likelihood of F-days in autumn compared to male patients (OR = 6.452, 95% CI: 1.651-25.20, P = 0.007). Age (OR = 1.036, 95% CI: 0.976-1.099, P = 0.23) and BMI (OR = 0.839, 95% CI: 0.672-1.049, P = 0.148) were not significantly associated with F-days in autumn.

**Table 10:** Factors associated with F-days in autumn (multivariate logistic regression analysis) F-days: Days with ≥3 AMI cases OR: Odds Ratio; CI: Confidence Intervals; AMI: Acute Myocardial Infarction

Variables	OR (95% CI)	P value
Sex	6.452 (1.651-25.20)	0.007
Age	1.036 (0.976-1.099)	0.23
BMI	0.839 (0.672-1.049)	0.15

## Discussion

This research emphasizes the seasonal variation in the incidence of AMI in Oita Prefecture. During the summer, elevated atmospheric pressure on the day before and two days before onset (day-1 and day-2) was associated with an increased incidence of AMI cases. In the autumn, on days with frequent AMI onset, patients were older and more likely to be female compared to days with non-frequent onset. The association between weather patterns and AMI appears to differ by region, likely influenced by variations in local climate. This was an observational study designed to explore associations and generate hypotheses regarding the influence of meteorological factors on AMI onset, rather than to establish definitive causal relationships.

In our registry, only non-F-days were observed during winter, as no F-days occurred in this season. Oita Prefecture belongs to a humid subtropical climate zone and experiences relatively mild winters. This seasonal absence of F-days may be partly explained by the region’s climatic characteristics. Previous studies have identified a link between the onset of AMI and maximum temperatures recorded two days before the event [7]. Reports from Kagoshima and Kumamoto Prefectures indicated relationships with temperature variations on day-1 and day-2 and low minimum temperatures on day-2 before onset [8,11]. In contrast, the present study in Oita Prefecture suggests that high atmospheric pressure on day-1 and day-2 prior to AMI onset, rather than temperature, may be related to AMI occurrence. These discrepancies may be attributable to differences in geographical and climatic conditions, as well as lifestyle or population characteristics.

While low atmospheric conditions reportedly increase AMI incidence [12], pressure increases or changes could also be related to AMI [4,13]. Danet et al. reported a V-shaped relationship between atmospheric pressure and AMI onset [14]. Our research found that high atmospheric pressure over one to two days correlated with the frequency of AMI. Additionally, fluctuations in atmospheric pressure may play a role in the emergence of ruptured plaques [4,15]; however, the mechanisms remain unclear. Regarding the effect of atmospheric pressure on blood pressure (BP), Weinbacher et al. reported a negative correlation between atmospheric pressure and systolic BP [16]. Conversely, other reports found no correlation between atmospheric pressure and elevated BP [13,17]. Charach et al. observed significant BP fluctuations in individuals aged 65-75 under 1007-1024 hPa [18]. High atmospheric pressure during summer may induce BP fluctuations, potentially contributing to an increased incidence of AMI. In addition to meteorological factors, seasonal variations in health behaviors may influence AMI onset. For example, Chaloupka reported that cigarette sales peaked in summer and were lowest in winter between 1983 and 1997 [19], suggesting a possible role of lifestyle changes in cardiovascular risk.

AMI onset may be more sensitive to weather in younger individuals than the elderly [6]. However, mortality rates due to low temperatures are higher in elderly individuals, particularly in older women exposed to cold environments [14]. Blood pressure fluctuations are associated with age and BMI, being more pronounced in individuals aged 65-75 years than in those over 75 [18]. Elderly individuals may experience this difference because of a compromised autonomic nervous system. Additionally, women’s physiological traits play a role in their risk. Estrogen’s anti-atherosclerotic properties help explain why premenopausal women have a lower incidence of coronary artery disease. After menopause, the reduction in estrogen eliminates this protective factor, resulting in a heightened risk of AMI among older women [14,20]. The reasons for the increased incidence of AMI in women during autumn remain unclear. However, it is hypothesized that women are more susceptible to temperature fluctuations than men, with elderly women being particularly sensitive to cold exposure [21]. Their relatively lower muscle mass may impair thermoregulation and heat production [11], making it more difficult to adapt to cold environments. In addition, cold-induced vasoconstriction, along with increases in blood pressure and blood viscosity, may further elevate the risk of AMI in this population [11,22]. Conversely, high temperatures can cause dehydration, which also contributes to blood thickening and increased cardiovascular risk [16,22]. Although these mechanisms are biologically plausible, our data do not directly support them. As the aging of Japanese women progresses, an increase in the incidence and mortality of AMI with advancing age has been reported. If weather conditions can be used as indicators to predict AMI onset, it may enable early detection and prompt medical intervention, potentially reducing the risk of severe outcomes and mortality.

We verified that elevated atmospheric pressure in summer correlated with a higher incidence of AMI, while in autumn, older adults and women faced an increased risk of AMI. These results emphasize the role of seasonal and meteorological elements in the onset of AMI, indicating the necessity for tailored preventive strategies by region. Furthermore, monitoring blood pressure, including patient education on accurate self-measurement and recording, and ensuring a consistent living environment, could help reduce the risk of AMI among at-risk groups, particularly during harsh weather conditions.

Limitations

This study has several important limitations. First, the meteorological data were derived from a single weather station in Oita City, which may not fully capture geographic variability across the prefecture. However, since most AMI cases in the registry occurred in Oita City and Beppu City, the meteorological conditions are likely representative of the central and eastern regions of Oita Prefecture. Second, the observation period of the Oita AMI Registry was relatively short (1.5 years), which may have limited our ability to identify long-term or inter-annual trends.

Third, the number of F-days was limited, particularly in summer (n=9) and autumn (n=6), and no F-days occurred in winter. These small sample sizes may have reduced the statistical power and limited the generalizability of seasonal subgroup analyses. The small sample sizes in some F-day groups may have limited the statistical power.

Additionally, while atmospheric pressure was significantly elevated on F-days during summer, this finding is not clearly supported by biological plausibility or consistent seasonal patterns.

Finally, although we adjusted for clinical and meteorological variables in our multivariable analysis, unmeasured confounding remains possible. Factors such as comorbidities, medication use, and seasonal variations in health behaviors (e.g., smoking, physical activity) were not fully captured in this registry and may have influenced the observed associations.

## Conclusions

Our research indicated that elevated atmospheric pressure during summer was associated with a higher occurrence of AMI on days with frequent onset of cardiac events. Although no significant meteorological factors were associated with AMI onset in autumn, female patients were independently associated with a higher occurrence of AMI on such days.

These findings highlight the need for region-specific preventive strategies, including the management of blood pressure fluctuations due to atmospheric changes and the promotion of a stable living environment. Further research is needed to clarify underlying mechanisms and develop effective interventions.
